# Risk Factors for Infectious Diseases in Backyard Poultry Farms in the Poyang Lake Area, China

**DOI:** 10.1371/journal.pone.0067366

**Published:** 2013-06-20

**Authors:** Yong Wang, Zhiben Jiang, Zhenyu Jin, Hua Tan, Bing Xu

**Affiliations:** 1 School of Environment, Tsinghua University, Beijing, China; 2 College of Global Change and Earth System Science, Beijing Normal University, Beijing, China; 3 Department of Geography, University of Utah, Salt Lake City, Utah, United States of America; The Australian National University, Australia

## Abstract

Emergence and transmission of infectious diseases have an enormous impact on the poultry industry and present a serious threat to the health of humans and wild birds. Noncommercial poultry operations, such as backyard poultry facilities in China, are potential sources of virus exchange between commercial poultry and wild birds. It is particularly critical in wetland areas where backyard poultry have close contact with commercial poultry and migratory birds, therefore increasing the risk of contracting infectious diseases. To evaluate the transmission risks, a cross-sectional study was undertaken in the Poyang Lake area, China, involving 309 residents in the backyard poultry farms in three counties (Region A, B, and C) of Jiangxi Province. We examined the backyard poultry population, poultry species, presence of poultry deaths from infectious diseases, food sources, and biosecurity practices. Region B ranked highest for biosecurity while region C ranked lowest. The risks of infectious diseases were assessed by adjusted odds ratio based on multivariate logistic regression analysis. Potential risk factors in the three regions of the study site were compared. In Region A, significant factor was contact of poultry with wild birds (OR: 6.573, 95% CI: 2.148–20.115, *P*=0.001). In Region B, the most significant factor was contact of poultry with neighboring backyard waterfowls (OR: 3.967, 95% CI: 1.555–10.122, *P*=0.004). In Region C, significant factors were poultry purchase from local live bird markets (OR: 3.740, 95% CI: 1.243–11.255, *P*=0.019), and contact of poultry with wild birds (OR: 3.379, 95% CI: 1.058–10.791, *P*=0.040). In summary, backyard poultry was significantly affected by neighboring commercial poultry and close contact with wild birds. The results are expected to improve our understanding of the transmission risks of infectious diseases in a typical backyard poultry environment in rural China, and address the need to improve local farming practices and take preventive measures.

## Introduction

China is one of the world primary producers of poultry products, and poultry is one of the main sources of meat in the country [1]. Poultry production generates 16 million tons of meat and 27 million tons of eggs annually in China [1]. The economic impact of infectious diseases on the poultry industry is significant. In 2004, the total loss caused by the highly pathogenic avian influenza virus (HPAIV) H5N1 was 4.5 billion US dollars in China [2]. Husbandry practices including biosecurity measures, poultry species and rearing scale vary between different poultry raising systems [3], and understanding these practices is important for implementing preventive measures.

Therefore, risk factors that affect the dynamics of infectious disease transmission between different birds and across bird species, need to be identified. Wild waterfowl is recognized as a natural host for influenza A viruses [4–6]. The interaction of domestic poultry with other animals, particularly wild birds, has been recognized as a possible source of avian disease in domestic flocks [7]. It may also allow the disease to be transmitted from domestic poultry back to the wild bird populations. Some production practices, such as free-ranging may increase the chance of such an interaction and therefore the risk of disease transmission [8,9]. Although the role that migratory birds play in the transmission of the current HPAIV H5N1 to domestic poultry is unclear, phylogenetic comparison of the viruses obtained from different hosts suggest that migratory birds carry the virus and spread it to domestic birds [10,11].

The transportation of poultry or working utensils may facilitate the transmission and spread of avian diseases [12,13]. In particular, transportation via live bird markets is believed to have played an instrumental role in several outbreaks of avian diseases that have caused significant economic loss in the United States [14–16]. The spread of avian diseases has also been associated with the movement of humans between different bird flocks [17–19].

Husbandry practices in China’s poultry-producing systems have not been fully examined in previous studies. From our observation, there are two distinct systems for commercial production and backyard poultry keeping. Local veterinarian told us that commercial farms usually operate on a large scale and poultry is generally kept in high density and enclosed housing conditions with high biosecurity measures. On the contrary, the majority of backyard poultry owners raises the poultry in open yards in a small scale, and do not apply much biosecurity measures. The flocks are raised for owners’ consumption only.

The HPAIVs that have been isolated from backyard flocks [16,20] and free range poultry are usually associated with the transmission of the lowly pathogenic avian influenza virus from wild birds [7,21]. The exotic Newcastle disease outbreak that occurred between 2002 and 2003 in the United States started among backyard poultry flocks and subsequently spread to commercial flocks [22]. An improved understanding of the risk factors for the backyard operation will help to control the transmission of infectious diseases and implement preventive strategies.

Poyang Lake area was selected as the study area because it is the largest freshwater lake in China with a total water area of more than 4,000 km^2^ in the summer wet season and less than 300 km^2^ during winter dry months [23]. Over 520,000 wild birds were found at the Poyang Lake during the wintering period in 2006 [24].

The objectives of this epidemiological survey were to acquire information on the biosecurity procedures adopted by backyard poultry growers, the number and type of birds raised, and the husbandry practices; and to identify risk factors that are associated with infections in backyard poultry in rural China.

## Materials and Methods

### Ethics statement

The protocol of the study was approved by the institutional ethics committee in the School of Environment, Tsinghua University. Prior to interview, a printed document detailing objective and format of the study was given to each participant. Signed consent was obtained from all participants prior to interview.

### Survey locations

**Figure 1:**
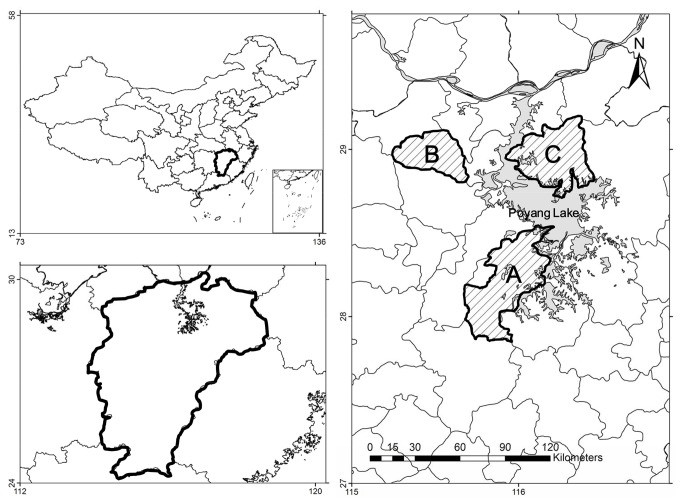
Study area around Poyang Lake of Jiangxi Province. A: Region A with 8.5 million laying ducks, B: Region B with 4 million laying hens, C: Region C without any commercial poultry farms.

Three counties were selected around Poyang Lake for the study (Figure 1): Nanchang County (Region A), De’an County (Region B) and Duchang County (Region C). These three regions were chosen for a comparative study because they represented different backyard poultry farming with different economic development and presence of commercial poultry industry.

Region A is located in the southern part of Poyang Lake, and close to the Nanji Wetlands National Nature Reserve. The annual net income of local farmers was 1,182 USD per person in 2010. Until the end of 2010, the whole county had 8.5 million laying ducks, ranking the highest in the Poyang Lake area [25]. Region B is located in the western part of Poyang Lake, and close to the Poyang Lake National Nature Reserve. The annual net income was 909 USD per person in 2010. Until the end of 2010, the whole county had 4 million laying hens, ranking the highest in the Poyang Lake area [25]. Region C is located in the northern part of Poyang Lake, and close to the Duchang Migratory Birds Provincial Nature Reserve. The annual net income of farmers was 554 USD per person in 2010 [25]. There was no commercial farm with more than 300 birds in this area according to the local veterinary services,

### Questionnaires

A cross-sectional survey was conducted to investigate rearing and biosecurity practices, and flock health history. A draft questionnaire was designed and pretested at thirteen farms. This draft was modified based on the results obtained from the pretest. The translated questionnaire is provided in the supporting information (Text S1).

The final questionnaire consisted of three sections. Section 1 referred to the demographic features and characteristics of the poultry species and farm size. Section 2 involved biosecurity practices of the household including poultry raising style, routine trading behavior, vaccination procedure, and contact with wild birds. Section 3 acquired information on flock health history. In the questionnaire, “contact with neighboring poultry” is defined as sharing foraging with neighboring flocks. “Accessibility to wild birds” is defined as presence of wild birds in the activity space of poultry except for sharing feed. “Contact with wild birds” is defined as sharing foraging with wild birds. “Free ranging” indicates no restriction on flocks’ foraging activity in the daytime.

Before the survey was conducted among the residents in the villages, we exchanged information with local veterinarians in the county government and familiarized ourselves with the basic conditions of backyard poultry rearing. The local government would organize free immunizations twice a year. The only vaccine that local residents are required to give the backyard flocks is H5 subtype avian influenza. Local veterinarians pointed out that the prevailing infectious diseases were avian influenza, Newcastle disease, *Escherichia coli* infection, fowl cholera and egg drop syndrome. In addition, several strains of avian influenza virus and Newcastle disease virus have been previously isolated from the backyard poultry flocks in the local area. When multiple unexplained deaths of backyard poultry were reported to the local government, veterinarians would be sent to conduct a necropsy to determine cause of death and advise appropriate control measures.

### Data collection

Region A was first divided into 18 townships, each consisting of 10–31 administrative villages, and each administrative village comprised several natural villages. The addresses of the commercial and backyard poultry farms were collected from local livestock offices. Based on this information, we then chose three townships with the highest density of commercial poultry farming. Within each administrative village, two natural villages were randomly sampled. Every natural village was assigned with a unique number. In each selected natural village, the number of households was finally chosen in proportion to the number of poultry-raising farms. A total of 83 backyard farms were included in Region A. Similarly, 157 backyard farms were selected for Region B. There was no commercial poultry farming in Region C, so the two townships in Region C were chosen randomly. A total of 69 backyard farms were selected from Region C by the same sampling method used in Region A. Backyard poultry owners were initially contacted by local village leader, informed of the purpose and format of the study, and made appointments for interviews. If the poultry owners were absent, the questionnaires would be completed by an adult (≥ 18 years) family member who helped with raising the poultry. The questionnaire was administered by three trained individuals who interviewed farm owners or an adult family member if the owner was absent. Among a total of 309 questionnaires, only 15 were not completed by poultry owners. The questionnaires were maintained by the investigators and would not be released to the third party for confidentiality.

### Quality control and statistical analysis

The questionnaire data were checked, errors in data entry were corrected, and completeness was evaluated. To cross check the data entry, two individual interviewers entered twice the data into EpiData V3.1 (EpiData, Odense, Denmark) and then analyzed them in S+ 8.2 (TIBCO, Palo Alto, CA, USA). Data distributions were examined across study strata, including density of commercial poultry farms and species of commercial poultry. Region A differs from Region B in that the major commercial poultry in Region A is free ranging laying ducks, while the major commercial poultry in Region B is captive laying hens. Therefore, Region A is compared with Region B in terms of species of commercial poultry and rearing style. Region A+B differs from Region C in that both Region A and Region B own large scale commercial poultry farms in high densities, while Region C does not own any large scale commercial poultry farms. Therefore, Region A+B is compared with Region C in terms of scale or density of commercial poultry farms. Mann–Whitney tests were performed to compare between regions for ranking data. chi-square (χ^2^) or Fisher’s exact tests were performed for counting data obtained from survey.

Flock health history data were acquired to design a case–control study. Univariate analysis was conducted to test for associations between presence of disease mortality and health history by using χ^2^ tests for categorical variables. To examine the independence of explanatory variables, multivariate logistic regression analysis was performed. Variables from univariate analyses with *P* values < 0.10 were included in the multivariate regression. The final model of potential risk factors was constructed by stepwise backward selection applying the iterative maximum-likelihood estimation procedure. Adjusted odds ratios (ORs) and 95% confidence intervals (CIs) were calculated. A *P* value < 0.05 was considered statistically significant.

## Results

A total of 62 villages in Poyang Lake area were included in this study. Three hundred and nine backyard poultry owners completed their questionnaires.

### Characteristics of backyard poultry flocks

A majority of families owned chicken only (82.20%) in their backyards. Some households raised more than one species of poultry, for example, chicken and duck (13.27%). Most flock size was less than 10 for chicken (161/233), duck (38/49) and goose (6/7). Most of the growers raised backyard birds as a source of food for family use, and only 2.91% of growers (6.02% of growers in Region A, 2.55% of growers in Region B, and none in Region C) reported selling their birds to dealers in all three regions.

The data summarized in Table 1 illustrate responses to questions pertaining to the flock characteristics of three regions, including species, number and proportion of poultry. There was a significant difference between Region A and Region B in terms of species of poultry raised in the backyard (*P* < 0.001). The percentage of chickens was only 51.81% in Region A versus 91.10% in Region B. The percentage of farmers who raised both chickens and ducks was 38.55% in Region A, while it was only 5.70% in Region B. Comparison between Regions A+B and Region C is as follows: 77.50% of the farms in Regions A+B raised chickens alone, while this percentage was 98.55% in Region C; and 17.08% of the farms in Regions A+B raised duck and chickens but no farms did this in Region C. There were significant differences in chicken capacity per farm between Region A and Region B (*P* = 0.014). The capacity was < 10 chicken in 44.16% of farms in Region A versus 59.62% in Region B. Only two chicken farms had > 50 birds and two duck farms had > 25 birds in three regions. Raising geese was not common (three farms in Region A, four in Region B, and one in Region C).

**Table 1:** Descriptive analysis of species, number, purpose and proportion (%) of poultry included in survey backyard farms in Jiangxi, China.

		**A+B vs** C^a^			**A vs** B^b^		
**Characteristic**		**A+B (%)**	**C (%)**	*P* ^c^	**A (%)**	**B (%)**	*P* ^c^
Species of poultry*		n=240	n=69	<0.001	n=83	n=157	<0.001
	Chicken only	186 (77.50)	68 (98.55)		43 (51.81)	143 (91.10)	
	Duck only	6 (2.50)	0 (0.00)		5 (6.00)	1 (0.60)	
	Goose only	0 (0.00)	0 (0.00)		0 (0.00)	0 (0.00)	
	Chicken+duck	41 (17.08)	0 (0.00)		32 (38.55)	9 (5.70)	
	Chicken+goose	5 (2.08)	1 (1.45)		2 (2.41)	3 (1.90)	
	Duck+goose	1 (0.42)	0 (0.00)		1 (1.20)	0 (0.00)	
	Chicken+duck+goose	1 (0.42)	0 (0.00)		0 (0.00)	1 (0.60)	
Number of birds*							
Chicken		n=233	n=69	0.830	n=77	n=156	0.014
	1-10	127 (54.51)	34 (49.28)		34 (44.16)	93 (59.62)	
	11-25	83 (35.62)	33 (47.83)		31 (40.26)	52 (33.33)	
	26-50	21 (9.01)	2 (2.90)		12 (15.58)	9 (5.77)	
	51-100	2 (0.86)	0 (0.00)		0 (0.00)	2 (1.28)	
Duck		n=49	n=0		n=38	n=11	0.046
	1-10	38 (77.55)	0 (0.00)		27 (71.05)	11 (100.00)	
	11-25	9 (18.37)	0 (0.00)		9 (23.68)	0 (0.00)	
	26-50	2 (4.08)	0 (0.00)		2 (5.26)	0 (0.00)	
Goose		n=7	n=1	0.705	n=3	n=4	0.248
	1-10	6 (85.71)	1 (100.00)		2 (66.67)	4 (100.00)	
	11-25	1 (14.29)	0 (0.00)		1 (33.33)	0 (0.00)	
Purpose of raising**		n=240	n=69		n=83	n=157	
	Source of food	240 (100.00)	69 (100.00)	1.000	83 (100.00)	157 (100.00)	1.000
	Source of income	9 (3.75)	0 (0.00)	0.103	5 (6.02)	4 (2.55)	0.178

^a^ Comparing penned commercial poultry (Regions A+B) versus Region C with no commercial poultry. ^b^ Comparing penned laying ducks (Region A) versus penned laying hens (Region B). ^c^ χ^2^ or Fisher’s exact tests. * Exclusive. ** Non-exclusive. Individual question is annotated with exclusive or non-exclusive. If the question is exclusive, the percentages for the answers will sum up to 100%. If the question is non-exclusive, the percentages for the answers will not sum up to 100%.

### Contact between backyard poultry and other birds

Laying duck owners will always buy younger poultry and poultry feed from live bird market. This may promote indirect interaction between their backyard poultry and the live bird market. However, a large number of farmers (90.61%) did not buy any food from wet market for their backyard poultry. Wet market and hatching themselves were two main sources of younger poultry, and only 18 (5.83%) owners reported that their younger poultry were obtained from other sources, such as commercial laying farms. There were 220 (71.20%) owners who reported presence of wild birds in the activity space of their backyard poultry. and 139 (44.98%) owners who reported that their backyard poultry shared foraging with wild birds. In addition, 242 (78.32%) owners reported that their backyard poultry shared foraging with neighboring poultry flocks.

Table 2 summarizes the answers to questions regarding the direct contact, accessibility of backyard poultry with other birds, and indirect disease transmission sources, such as live bird market. There was no obvious difference in the source of younger poultry between the regions, except that the growers obtained younger domestic birds from the market more frequently in Region A than in Region B; similarly, it was more frequent in Regions A+B than in Region C (*P*=0.006). Likewise, the growers obtained feed for poultry from the market more frequently in Region A than in Region B (*P*=0.013). Similarly, in Regions A+B growers obtained poultry feed from the market more frequently than in Region C (*P*=0.002). The contact of backyard flocks in Region C with their neighboring chickens, 91.30%, was more frequent than that in Regions A+B (74.58%, *P* = 0.003), while the contact of backyard flocks in Region C with their neighboring waterfowls, 2.9%, was less frequent than that in Regions A+B (18.75%, *P* = 0.001). Wild birds were frequently observed in all three regions: 67.47% in Region A; 71.97% in Region B; and 73.91% in Region C, and there was no significant difference between these regions. In Region A, households reported a higher frequency of poultry contact with wild birds (73.21%) than that in Region B (49.59%, *P* = 0.004). And there was also a significant difference between Region C (82.35%) and Regions A+B (57.40%, *P* = 0.001).

**Table 2:** The source of younger poultry and feed, and contact between backyard poultry and other birds.

		**A+B vs C**			**A vs B**		
**Characteristic**		**A+B (%)**	**C (%)**	***P***	**A (%)**	**B (%)**	***P***
Younger poultry source**		n=240	n=69		n=83	n=157	
	Wet market	129 (53.75)	24 (34.78)	0.006	55 (66.27)	74 (47.13)	0.005
	Hatching themselves	132 (55.00)	41 (59.42)	0.515	41 (49.40)	91 (57.96)	0.206
Feed source**		n=240	n=69		n=83	n=157	
	Wet market	29 (12.08)	0 (0.00)	0.002	16 (19.28)	13 (8.28)	0.013
	Made by themselves	238 (99.17)	69 (100.00)	0.448	81 (97.59)	157 (100.00)	0.051
Contact with neighboring poultry**		n=240	n=69		n=83	n=157	
	Chicken	179 (74.58)	63 (91.30)	0.003	57 (68.67)	122 (77.71)	0.127
	Waterfowl	45 (18.75)	2 (2.90)	0.001	20 (24.10)	25 (15.92)	0.124
	None	61 (25.42)	6 (8.70)	0.003	26 (31.33)	35 (22.29)	0.127
Accessibility to wild birds*		n=240	n=69	0.573	n=83	n=157	0.468
	Yes	169 (70.42)	51 (73.91)		56 (67.47)	113 (71.97)	
	No	71 (29.58)	18 (26.09)		27 (32.53)	44 (28.03)	
Contact with wild birds*		n=169	n=51	0.001	n=56	n=113	0.004
	Yes	97 (57.40)	42 (82.35)		41 (73.21)	56 (49.56)	
	No	72 (42.60)	9 (17.65)		15 (26.79)	57 (50.44)	

* Exclusive. ** Non-exclusive. Individual question is annotated with exclusive or non-exclusive. If the question is exclusive, the percentages for the answers will sum up to 100%. If the question is non-exclusive, the percentages for the answers will not sum up to 100%.

### Disease prevention measures and farming style

Among backyard flocks, 44.66% were not immunized. Among 171 vaccinated flocks, 68.42% were immunized by the government. There were 164 flocks that all poultry in their backyards completed their immunization, while only seven flocks were left for partial vaccination (9.3% in Region A, 2.91% in Region B, and none in Region C). Growers explained that vaccination might affect egg production. A great number of owners (88.35%) raise backyard poultry every year, and 89.97% of the owners kept their poultry in a free ranging style.

There was no difference in the frequency of vaccination between Region A and Region B, but the growers in Regions A+B reported more frequent vaccination than those in Region C (Table 3). The vaccination rate in Regions A+B was significantly higher than in Region C (*P*<0.001). In Region B, growers reported a higher frequency of vaccination by government (87.38%), while in Region A, vaccination by government was uncommon, and a significantly larger number of growers reported that they vaccinated their birds by themselves (86.05%, *P*<0.001). The households raised backyard poultry more frequently every year in Region A (96.39%) than those in Region B did (81.89%, *P*=0.001), and there was also a difference between Regions A+B (86.25%) and Region C (95.65%, *P*=0.032). There was a significant difference in the proportion of free ranging between Region A (96.39%) and Region B (88.54%, *P*=0.041), but no significant difference between Regions A+B and Region C (*P*=0.162).

**Table 3:** Characteristics of vaccination and raising practice.

		**A+B vs C**			**A vs B**		
**Characteristic**		**A+B (%)**	**C (%)**	***P***	**A (%)**	**B (%)**	***P***
Frequency of vaccination*		n=240	n=69	<0.001	n=83	n=157	0.172
	0	94 (39.17)	44 (63.77)		40 (48.19)	54 (34.39)	
	1	60 (25.00)	18 (26.09)		20 (24.10)	40 (25.48)	
	2	69 (28.75)	7 (10.14)		9 (10.84)	60 (38.22)	
	≥3	17 (7.08)	0 (0.00)		14 (16.87)	3 (1.91)	
Vaccinated by*		n=146	n=25	0.071	n=43	n=103	<0.001
	Government	96 (65.75)	21 (84.00)		6 (13.95)	90 (87.38)	
	Poultry owner	50 (34.25)	4 (16.00)		37 (86.05)	13 (12.62)	
Poultry vaccinated*		n=146	n=25	0.265	n=43	n=103	0.101
	All	139 (95.21)	25 (100.00)		39 (90.70)	100 (97.09)	
	Partly	7 (4.79)	0 (0.00)		4 (9.30)	3 (2.91)	
Raising frequency*		n=240	n=69	0.032	n=83	n=157	0.001
	Every year	207 (86.25)	66 (95.65)		80 (96.39)	127 (80.89)	
	Some years	33 (13.75)	3 (4.35)		3 (3.61)	30 (19.11)	
Type of raising*		n=240	n=69	0.162	n=83	n=157	0.041
	In captivity	21 (8.75)	10 (14.49)		3 (3.61)	18 (11.46)	
	Free range	219 (91.25)	59 (85.51)		80 (96.39)	139 (88.54)	

* Exclusive. Individual question is annotated with exclusive or non-exclusive. If the question is exclusive, the percentages for the answers will sum up to 100%. If the question is non-exclusive, the percentages for the answers will not sum up to 100%.

### Health history

Among the surveyed participants, 48.87% of owners reported backyard poultry mortality caused by infectious diseases in the past years. Eighty four owners reported deaths in the past 12 months (during the year of 2009), and all of the 84 owners also reported deaths in the previous 12 months (during the year before 2009). The growers in Region A reported a higher frequency of infectious disease (59.04%) than those in Region B (39.49%, *P*=0.004), but there was no significant difference between Regions A+B and Region C (Table 4).

**Table 4:** Health history in the past two years.

		**A+B vs C**			**A vs B**		
**Characteristic**		**A+B (%)**	**C (%)**	***P***	**A (%)**	**B (%)**	***P***
Infectious disease death*		n=240	n=69	0.087	n=83	n=157	0.004
	Yes	111 (46.25)	40 (57.97)		49 (59.04)	62 (39.49)	
	No	129 (53.75)	29 (42.03)		34 (40.96)	95 (60.51)	
The onset of infection disease death*		n=111	n=40	0.308	n=49	n=62	0.986
	Death occurs only in the last 12 months	0 (0.00)	0 (0.00)		0 (0.00)	0 (0.00)	
	Death occurs only in 12 months ago	52 (46.85)	15 (37.50)		23 (46.94)	29 (46.77)	
	Death occurs in both time	59 (53.15)	25 (62.50)		26 (53.06)	33 (53.23)	

*Exclusive. Individual question is annotated with exclusive or non-exclusive. If the question is exclusive, the percentages for the answers will sum up to 100%. If the question is non-exclusive, the percentages for the answers will not sum up to 100%.

### Potential risk factors

Characteristics of backyard poultry rearing practices differ in three regions. Region A owns the most free-ranging laying ducks, with poor completion of required vaccination. The most common source for younger poultry is from the live bird market. The backyard poultry in this region has relatively high contact rate with wild birds and neighboring waterfowls. Region B has the largest scale of commercial production of laying hens with highest biosecurity measures, such as regular disinfection, timely vaccination, monitored transportation of products, etc. Region C has the lowest annual income per person with worst vaccination completion. Backyard poultry in this region has the highest contact rate with neighboring poultry and wild birds, and highest accessibility to wild birds. The poultry mortality caused by infectious diseases ranks the lowest in region B and high in both Region A and C. Implications for different disease control strategies due to regional differences will be suggested and discussed in terms of these known factors for disease spread. These known factors are considered as potential risk factors for infectious diseases in backyard poultry in three different regions and analyzed through univariate analysis by a case control study design.

Logistic regression was applied to model backyard poultry deaths caused from infectious diseases in 2009 and potential risk factors. Table 5 shows the results of the univariate analyses in Region A. In this region, “poultry contact with wild birds” (OR: 5.714, 95% CI: 1.981–16.481, *P*=0.001); and “poultry contact with neighboring backyard waterfowls” (OR: 2.937, 95% CI: 1.034–8.345, *P*=0.043) were significantly associated with infection (*P* ≤ 0.05) by univariate analysis. Table 6 shows the results of the univariate analyses in Region B. In this region, the factors positively associated with case farms by univariate analysis were: “raising more than one kind of poultry” (OR: 5.295, 95% CI: 1.643–17.063, *P*=0.005); “poultry contact with neighboring backyard waterfowls” (OR: 4.879, 95% CI: 1.959–12.155, *P*=0.001); and “farm accessible to wild birds” (OR: 3.452, 95% CI: 1.136–10.488, *P*=0.029). Three variables were considered for logistic regression. Table 7 shows the results of the univariate analyses in Region C. In this region, “buy younger poultry from wet market” (OR: 3.250, 95% CI: 1.149–9.193, *P*=0.026); and “poultry contact with wild birds” (OR: 2.891, 95% CI: 0.970–8.616, *P*=0.057) were the two significant risk factors by univariate analysis.

**Table 5:** Univariate analysis of potential risk factors for infectious disease in backyard poultry in Region A (Nanchang Country), Poyang Lake area, Jiangxi, China, 2009.

		**Case farms**	**Control farms**		
**Characteristic**		**n=26, n (%)**	**n=57, n (%)**	**OR** (**95% CI**)^a^	***P* value**
	Raising more than one species of poultry	12 (46.15)	23 (40.35)	1.267 (0.497-3.228)	0.602
	Free range	25 (96.15)	55 (96.49)	0.909 (0.079-10.449)	0.939
	Buy younger poultry from wet market	19 (73.08)	36 (63.16)	1.583 (0.571-4.391)	0.377
	Buy poultry food from wet market	2 (7.69)	14 (24.56)	0.256 (0.054-1.222)	0.088
	Raising for income	2 (7.69)	3 (5.26)	1.500 (0.235-9.565)	0.668
	Vaccinated by government	4 (15.38)	2 (3.51)	5.000 (0.853-29.293)	0.074
	Vaccinated by poultry owners	7 (26.92)	30 (52.63)	0.332 (0.121-0.911)	0.032
	All vaccinated	10 (38.46)	29 (50.88)	0.603 (0.234-1.553)	0.295
	Partly vaccinated	1 (3.85)	3 (5.26)	0.720 (0.071-7.270)	0.781
	Poultry contact with neighboring backyard chickens	21 (80.77)	36 (63.16)	2.450 (0.804-7.463)	0.115
	Poultry contact with neighboring backyard waterfowls	10 (38.46)	10 (17.54)	2.937 (1.034-8.345)	0.043
	Farm accessibility to wild birds	21 (80.77)	35 (61.40)	2.640 (0.869-8.023)	0.087
	Poultry contact with wild birds	20 (76.92)	21 (36.84)	5.714 (1.981-16.481)	0.001

^a^ OR, odds ratio; CI, confidence interval

**Table 6:** Univariate analysis of possible risk factors for infectious disease in backyard poultry in Region B (De’an Country), Poyang Lake area, Jiangxi, China, 2009.

		**Case farms**	**Control farms**		
**Characteristic**		**n=33, n (%)**	**n=124, n (%)**	**OR (95% CI)**	***P* value**
	Raising more than one species of poultry	7 (21.21)	6 (4.84)	5.295 (1.643-17.063)	0.005
	Free range	29 (87.88)	110 (88.71)	0.923 (0.282-3.015)	0.894
	Buy younger poultry from wet market	18 (54.55)	53 (42.74)	1.608 (0.743-3.479)	0.228
	Buy poultry food from wet market	3 (9.09)	10 (8.06)	1.140 (0.295-4.404)	0.849
	Raising for income	1 (3.03)	3 (2.42)	1.260 (0.127-12.527)	0.843
	Vaccinated by government	18 (54.55)	72 (58.06)	0.867 (0.400-1.876)	0.717
	Vaccinated by poultry owners	2 (6.06)	11 (8.87)	0.663 (0.140-3.148)	0.605
	All vaccinated	20 (60.61)	80 (64.52)	0.846 (0.384-1.863)	0.678
	Partly vaccinated	0 (0.00)	3 (2.42)	Incalculable	NA
	Poultry contact with neighboring backyard chickens	30 (90.91)	92 (74.19)	3.478 (0.993-12.178)	0.051
	Poultry contact with neighboring backyard waterfowls	12 (36.36)	13 (10.48)	4.879 (1.959-12.155)	0.001
	Farm accessibility to wild birds	29 (87.88)	84 (67.74)	3.452 (1.136-10.488)	0.029
	Poultry contact with wild birds	13 (39.39)	43 (34.68)	1.224 (0.556-2.698)	0.616

**Table 7:** Univariate analysis of possible risk factors for infectious disease in backyard poultry in Region C (Duchang Country), Poyang Lake area, Jiangxi, China, 2009.

		**Case farms**	**Control farms**		
**Characteristic**		**n=25, n (%)**	**n=44, n (%)**	**OR (95% CI)**	***P* value**
	Raising more than one species of poultry	0 (0.00)	1 (2.27)	Incalculable	NA
	Free range	20 (80.00)	39 (88.64)	0.513 (0.133-1.982)	0.333
	Buy younger poultry from wet market	13 (52.00)	11 (25.00)	3.250 (1.149-9.193)	0.026
	Buy poultry food from wet market	0 (0.00)	0 (0.00)	Incalculable	NA
	Raising for income	0 (0.00)	0 (0.00)	Incalculable	NA
	Vaccinated by government	7 (28.00)	14 (31.82)	0.833 (0.283-2.452)	0.741
	Vaccinated by poultry owners	0 (0.00)	4 (9.09)	Incalculable	NA
	All vaccinated	7 (28.00)	18 (40.91)	0.562 (0.195-1.621)	0.286
	Partly vaccinated	0 (0.00)	0 (0.00)	Incalculable	NA
	Poultry contact with neighboring backyard chickens	23 (92.00)	40 (90.91)	1.150 (0.195-6.773)	0.877
	Poultry contact with neighboring backyard waterfowls	1 (4.00)	1 (2.27)	1.792 (0.107-29.952)	0.685
	Farm accessibility to wild birds	19 (76.00)	32 (72.73)	1.187 (0.383-3.685)	0.766
	Poultry contact with wild birds	19 (76.00)	23 (52.27)	2.891 (0.970-8.616)	0.057

Multivariate analysis of potential risk factors was performed combining all three regions (Table 8). In Region A, six variables with *P* ≤ 0.1 were considered for inclusion in the final multivariate logistic regression model to estimate independence of effects. The final logistic regression model included one variable as an independent risk factor for disease death: “poultry contact with wild birds” (OR: 6.573, 95% CI: 2.148–20.115, *P*=0.001). In Region B, “poultry contact with neighboring backyard waterfowls” was independently and positively associated with infection (OR: 3.967, 95% CI: 1.555–10.122, *P*=0.004). In Region C, two factors were identified as independent risk factors in the final model: “buy younger poultry from wet market” (OR: 3.740, 95% CI: 1.243–11.255, *P*=0.019); and “poultry contact with wild birds” (OR: 3.379, 95% CI: 1.058–10.791, *P*=0.040).

**Table 8:** Result of multivariate analysis of potential risk factor for backyard poultry death of infectious disease, Poyang Lake area, Jiangxi, China, 2009.

**Characteristic**		**Odds ratio**	**95% CI**	***P* value**
**Region A**				
	Poultry contact with wild birds	6.573	2.148-20.115	0.001
**Region B**				
	Poultry contact with neighboring backyard waterfowls	3.967	1.555-10.122	0.004
**Region C**				
	Buy younger poultry from wet market	3.740	1.243-11.255	0.019
	Poultry contact with wild birds	3.379	1.058-10.791	0.040

## Discussion and Conclusion

A combination of descriptive and analytical epidemiological techniques was used to explore and evaluate potential risk factors associated with mortality of backyard poultry caused by infectious diseases in the Poyang Lake area of China.

It is summarized from the survey that the frequency of waterfowl raising in Region A is higher than that in Region B and Region C. Region A is in plain areas with dense river networks, which are conducive to the raising of waterfowls. Region B and Region C were in relatively hilly areas. Previous studies have recognized domestic ducks as a risk factor for disease occurrence owing to their potential role as a reservoir of infectious diseases [26–29]. The riparian zones of the lake provide major food sources including vegetation, fish and crustaceans for waterfowl. The water body can be contaminated by congregating sick ducks [30,31]. Backyard poultry may be exposed to virus transmission while they share the same water area.

Wet markets are considered to be an important source of infection [32]. Locally produced poultry is mixed with imported poultry for more than a day, thus viruses may be amplified in the markets [33]. Several subtypes of AIVs have previously been isolated from the markets in Poyang Lake area [34]. According to our interviews, no one purchased live adult poultry from markets and slaughtered them at home. A few people reported that they purchased live adult birds and had them slaughtered by the seller at the market several times annually. However, farmers do purchase younger poultry and poultry feed from live bird markets. Region A owns the most free-ranging laying ducks. The most common source for younger poultry is from the live bird market.

Viruses were found to be widely distributed among wild birds. Migratory waterfowl are particularly considered to be a natural reservoir of AIVs [5,35–37]. Resident birds have also been shown to harbor H5N1 AIVs [38,39]. Every winter, many migratory waterfowl inhabit along the riparian zones of Poyang Lake. This raises concern that migratory birds could introduce viruses into local resident wild birds, which might then deliver the virus to local flocks of domestic birds. Compared with those in Region B, the farmers in Region A and Region C reported higher frequency of backyard poultry contact with wild birds and neighboring poultry. The distance from Poyang Lake to Region B and the other two regions could be responsible for the difference in this risk factor.

Since 2005, H5N1 vaccination in China has been carried out by governmental veterinarians free of charge during two main seasons per year, March to April and October to November [40]. The difference in vaccination practices between Region A and other two regions showed that, self vaccination in Region A was organized to match with the production cycles of backyard poultry. However, the growers followed the governmental strategy of vaccinations twice in Region B and Region C. Our results indicate that vaccination and the timing of vaccination are important in making effective prevention strategies.

Most backyard poultry raised in the Poyang Lake area are in a free ranging style. This may increase the chance of poultry’s being exposed to neighboring poultry and wild birds, which will in turn increase the disease risk of backyard poultry. Backyard poultry farming is scattered. It would be difficult to catch the actual chain infection by a single cross-sectional survey. This survey recorded occurrence of poultry deaths caused by infectious disease in the past years. Additionally, in our previous study, several virus strains (1 strain of H5 subtype, 4 strains of H9 subtype of avian influenza virus and 3 strains of Newcastle disease virus) among a total of 1340 samples collected from backyard poultry in this area, were isolated using specific pathogen free (SPF) chicken embryos (unpublished data).

To reduce potential bias, standardized questionnaires and trained interviewers were provided. It was recognized that recall bias was likely to occur, particularly for questions on the date of death of backyard poultry and date of vaccination each year. In our pilot survey, we found that special days, such as the Chinese New Year/Spring Festival, could help growers to recall events more easily. The interview time was chosen near the Spring Festival, and the growers were asked to recall events between two consecutive Spring Festivals (within the last 12 months) and out with the previous 12 months.

Three remaining factors including poultry contact with neighboring backyard waterfowl, poultry contact with wild birds, and buying younger poultry from wet markets, were important for the spread and maintenance of infectious diseases in Poyang Lake area. However, potential risk varies from region to region, as characteristics of backyard poultry rearing practices differ from region to region. Scale of commercial free ranging laying ducks in Region A is largest. Younger poultry of laying ducks are mainly purchased from live bird markets. It is easily spotted that wild birds share feeds with the free ranging laying ducks. There is no doubt that the backyard poultry will have close contact with wild birds in this region. It is hard to reduce the contacts except for raising less backyard poultry or reducing the scale of free ranging laying ducks. The poultry mortality caused by infectious diseases in Region B ranks the lowest, although it has the largest scale of commercial production of laying hens, which requires high biosecurity measures. Although the amount of backyard waterfowls is small, “poultry contact with neighboring backyard waterfowl” is a significant risk factor. Farmers may further reduce the backyard waterfowl breeding. The economic situation of farmers in Region C ranks the poorest with worst vaccination completion and no commercial farms. Farmers should reduce buying younger poultry from live bird market, and reduce the chance of poultry contact with wild birds. To enforce biosecurity measures and implementation of immunization by means of education may be a practical way of improving the overall poultry rearing practices, thus reducing disease risks of backyard poultry.

Overall, the owners should be advised to reduce free-range rearing of poultry. During feeding, the owner may prevent wild birds from joining the flock, and the remaining feed in the poultry activity area must be removed. Villagers should reduce buying younger poultry from wet markets. Controls and restrictions in the transportation of live poultry should be enforced, such as safe transport of live poultry through clean and disinfected transport vehicles and cages, at the currently available border area check points.

Our results showed that live bird markets and wild birds were two potential risk factors of infectious diseases for backyard poultry in the Poyang Lake area. This suggests that backyard poultry in this area may serve as a disease intermediary between the live bird market and the wild bird. Therefore, avoidance of the risk factors identified in this study, and implementation of preventive measures, may reduce the risk for infectious diseases. Governmental agencies need to develop prevention and control strategies, adapting to local requirements to reduce poultry disease loss as much as possible. These findings can be generalized and applied to similar poultry rearing environment in other developing countries, particularly in Southeast Asia.

## Supporting Information Legends

Text S1 **The questionnaire of Poyang Lake backyard poultry (in English)**.
(DOCX)
