# Supplementary material for: Risk Factors for Infectious Diseases in Backyard Poultry Farms in the Poyang Lake Area, China
Source: PLoS One. 2013 Jun 20;8(6):e67366. doi: 10.1371/journal.pone.0067366 (PMC3688663; doi:10.1371/journal.pone.0067366)
Supplement: Text S1 — The questionnaire of Poyang Lake backyard poultry (in English). (DOCX) [file pone.0067366.s001.docx]

**The questionnaire of Poyang Lake backyard poultry**

Interview data： / / (Month/Day/Year)

Interviewer name:

**1. Basic information of poultry owner**

| Poultry owner name： | | Tel： |
| --- | --- | --- |
| The address of poultry owner: / / / (Country/ Township / Administrative village / Natural village) | | |
| GPS | Latitude: Longitude: [Elevation](http://dict.baidu.com/s?wd=elevation): | |

**2. Farm characterization**

| Total number of chicken owned (Fill the figures from 0 to 100) | _____ |
| --- | --- |
| Total number of duck owned (Fill the figures from 0 to 100) | _____ |
| Total number of goose owned (Fill the figures from 0 to 100) | _____ |
| The poultry were source of food (Binary) | □Yes □No |
| The poultry were source of income (Binary) | □Yes □No |

**3. Contact with neighboring poultry in the time of foraging for food**

| Own poultry contact with neighbor backyard chicken (Binary) | □Yes □No |
| --- | --- |
| Own poultry contact with neighbor backyard duck (Binary) | □Yes □No |
| Own poultry contact with neighbor backyard goose (Binary) | □Yes □No |

**4. Wild bird in poultry area**

| Observed wild bird in poultry area (Binary) (If the answer is “No”, we do not ask the following question in this table) | □Yes □No |
| --- | --- |
| Observed wild bird eat food with poultry (Binary) | □Yes □No |

**5. Vaccination measures (H5 vaccine)**

| Vaccinated in previous 12 months (Binary) (If the answer is “No”, we do not ask the following four questions in this table) | □Yes □No |
| --- | --- |
| Frequency of vaccinated in previous 12 months (Fill figures) | _____ |
| Vaccination by government (Binary) | □Yes □No |
| Vaccination by poultry owner (Binary) | □Yes □No |
| Rang of vaccinated (Binary) | □All poultry □Part of poultry |

**6. Other practices about backyard poultry raising**

| Obtained younger poultry from wet market (Binary) | □Yes □No |
| --- | --- |
| Obtained younger poultry by hatching themselves (Binary) | □Yes □No |
| Obtained poultry feed from wet market (Binary) | □Yes □No |
| Made poultry feed by themselves (Binary) | □Yes □No |
| Raising backyard poultry every year (Binary) | □Yes □No |
| Type of raising (Binary) (“Free range” refer to the poultry were not restricted of foraging process in the daytime) | □Free rang □In captivity |

**7. Health problems**

**7.1 In previous 12 months (If the death happened more than once, fill every death in a separate table)**

| Death happened in previous 12 months (Binary) (If the answer is “No”, we do not ask the following four questions in this table) | □Yes □No |
| --- | --- |
| The death happened data | / / (Month/Day/Year) |
| The death happened in neighboring backyard flock at the same time (Binary) | □Yes □No |
| The disease was diagnosed by a veterinarian (Binary) (If the answer is “No”, we do not ask the following question in this table) | □Yes □No |
| The veterinarian considered the Death by infectious poultry disease (Binary) | □Yes □No |

| Death happened in previous 12 months (Binary) (If the answer is “No”, we do not ask the following four questions in this table) | □Yes □No |
| --- | --- |
| The death happened data | / / (Month/Day/Year) |
| The death happened in neighboring backyard flock at the same time (Binary) | □Yes □No |
| The disease was diagnosed by a veterinarian (Binary) (If the answer is “No”, we do not ask the following question in this table) | □Yes □No |
| The veterinarian considered the Death by infectious poultry disease (Binary) | □Yes □No |

| Death happened in previous 12 months (Binary) (If the answer is “No”, we do not ask the following four questions in this table) | □Yes □No |
| --- | --- |
| The death happened data | / / (Month/Day/Year) |
| The death happened in neighboring backyard flock at the same time (Binary) | □Yes □No |
| The disease was diagnosed by a veterinarian (Binary) (If the answer is “No”, we do not ask the following question in this table) | □Yes □No |
| The veterinarian considered the Death by infectious poultry disease (Binary) | □Yes □No |

**7.2 In 12 months ago (If the death happened more than once, fill every death in a separate table)**

| Death happened in 12 months ago (Binary) (If the answer is “No”, we do not ask the following four questions in this table) | □Yes □No |
| --- | --- |
| The death happened data | / / (Month/Day/Year) |
| The death happened in neighboring backyard flock at the same time (Binary) | □Yes □No |
| The disease was diagnosed by a veterinarian (Binary) (If the answer is “No”, we do not ask the following question in this table) | □Yes □No |
| The veterinarian considered the Death by infectious poultry disease (Binary) | □Yes □No |

| Death happened in 12 months ago (Binary) (If the answer is “No”, we do not ask the following four questions in this table) | □Yes □No |
| --- | --- |
| The death happened data | / / (Month/Day/Year) |
| The death happened in neighboring backyard flock at the same time (Binary) | □Yes □No |
| The disease was diagnosed by a veterinarian (Binary) (If the answer is “No”, we do not ask the following question in this table) | □Yes □No |
| The veterinarian considered the Death by infectious poultry disease (Binary) | □Yes □No |

| Death happened in 12 months ago (Binary) (If the answer is “No”, we do not ask the following four questions in this table) | □Yes □No |
| --- | --- |
| The death happened data | / / (Month/Day/Year) |
| The death happened in neighboring backyard flock at the same time (Binary) | □Yes □No |
| The disease was diagnosed by a veterinarian (Binary) (If the answer is “No”, we do not ask the following question in this table) | □Yes □No |
| The veterinarian considered the Death by infectious poultry disease (Binary) | □Yes □No |
